# The Hon. Sir Arthur Stanley's Reply to "Ierne's" Open Letter

**Published:** 1918-06-01

**Authors:** Arthur Stanley


### WHY WORKING NURSES SHOULD ARTICULATE.

### The Hon. Sir Arthur Stanley's Reply to "Ierne's" Open Letter.

(See "The Hospital," May 18, 1918, page 143.)

Madam,—Criticism so friendly and so appreciative as that contained in your open letter to me, which appears in The Hospital of May 18, is encouraging and decidedly refreshing after the carping attacks upon the College of Nursing that have, up till now, taken the place of sound and helpful criticism and advice. Your letter is addressed to me personally, and it is as myself only, and not as Chairman of the College of Nursing, that I answer it.

Your main criticism is that we who are responsible for the policy of the College have reversed the correct order of procedure and that we have occupied our time wrangling about a Registration Bill when we should have been devoting our attention to the "economic problem." On the face of it, there would appear to be some justification for your criticism, but will you allow me to put the matter to you from my own point of view?

### A Uniform Standard of Training and Certificates.

When we took up the question of the organisation of the Nursing Profession, I and those working with me had foremost in our minds the desirability of securing a uniform standard of training and of certificate—in other words, our first idea was to safeguard the nurses themselves, and the general public, against the untrained or partially trained woman, who, as things are now, can get a certificate after three years' work in a hospital, or less, although she may never have received any training in the real sense of the word. That seemed to us to be the first problem that the College had got to tackle, and even in these early days I believe that we have gone far towards its solution. By "recognising" those training-schools whose standard of teaching and training comes up to the standard which we think right, and by refusing to recognise those training-schools whose teaching and training do not come up to that standard, the College has advanced an appreciable distance along the road which leads to the raising and maintaining of the standard of training, the promotion of a uniform curriculum, and the establishment of a one-portal examination—three of the objects which the College has set out to attain.

### "Things of Overwhelming Importance."

But as the idea of the College began to take shape and to grow, so our ideas grew with it, and we realised that the College, if it were really to represent the nursing profession, must take upon itself other duties and responsibilities. **We earnestly hoped that the nurses would turn to the College for help and would look to us—in your own words—"to ensure better conditions of service, better pay, shorter hours of duty, longer holidays, and, above all, to remove the constant anxiety of providing for age and infirmity." We agree with you in thinking that these things are of overwhelming importance, and with these objects in view we have fashioned the College in the way which, in our opinion, will facilitate their attainment.**

### Too Narrow a View.

One of the reasons why the various nursing associations have done so little for nurses in the past is, I believe, the fact that they have taken too narrow a view of the nurses' position. The nurse is now an integral and important factor in the whole social scheme, and to consider her apart from her environment is to set her in a false position—a female Athanasius against a whole world of governors and governing authorities. We realised that it was necessary to enlist the sympathy and to secure the support of those who govern the hospitals, and in this we have been far more successful than we anticipated. We have found the governors of civilian general hospitals and the Poor-Law Authorities ready, and indeed anxious, to meet us in consultation, and although little can be accomplished until the war is over, the spade-work which is being done now will, I am convinced, ensure a plentiful harvest in the days soon to come.

### The Nurses' Tribute Fund.

One item in your programme seemed to us to need dealing with at once. With the help of the British Women's Hospital we have started a fund which is destined to provide for those nurses who are in need, and we hope thus to tide over the time until, with the advent of better pay and better conditions, the nurse herself will be able to secure her own future, and the constant anxiety of providing for old age and infirmity will cease to exist.

### The Nurses Helping the College to Work.

I will not attempt to give an account in detail of all we have done and are doing to solve this economic problem, because we have, after all, only begun to deal with the vast amount of work that requires to be done. We are still young, although our growth—especially during the last few months—has been rapid, and it must be remembered that we had to construct the machine and to make certain that we had the necessary driving power in the nurses themselves before we could hope to turn out any finished work. Our machinery is now set up, the nurses are supplying the motive force in increasing volume every day, and we can now get to work. Write to me again two years hence—no, one year will be enough—and tell me whether you think we are fulfilling the promise of our early youth.

### Statutory Recognition Long Overdue.

As for the Registration Bill, speaking for myself alone and with all the respect that is due from a member of Parliament to that august assemblage, I hold much the same view as yourself. I believe that the statutory recognition of the nursing profession is necessary and is long overdue—indeed, it is the first plank of our College programme, but I have always tried to impress upon those audiences of nurses who have been kind enough to listen to me during the past three years that State Registration by itself cannot do much to create a new and better world for them.

How small of all that human hearts endure,
That part which laws or kings can cause or cure.

Before and After State Registration.

The passing of a Registration Bill will do little to help the nurses unless there is in existence a strong and united body ready to take hold of the Act and to carry out its provisions. That is why we of the College are not waiting until after the Bill is passed to set up the necessary machinery to work it; that is why we are calling to the nurses to join the College and thus to place their hand on the lever which controls the machinery, and that is why we are working hard to perfect our organisation so that when the long-looked-for day arrives and the Nurses' Registration Act stands upon the Statute Book we shall be ready to take the fullest advantage of the powers conferred upon us by the State. You will, I feel sure, forgive this disjointed reply to your own interesting letter. I have little time, and, I fear, even less ability to turn phrases and to polish periods, but I hope that you will see in what I have written my earnest wish to meet your friendly criticism in the spirit in which you offer it, feeling, as I do, that we are working each in our own way for the cause which we both have at heart—the welfare of the British Nurse.

Arthur Stanley.

